# Supplementary figures and images for: A novel murine model of post-implantation malaria-induced preterm birth
Source: PLoS One. 2022 Mar 21;17(3):e0256060. doi: 10.1371/journal.pone.0256060 (PMC8936457; doi:10.1371/journal.pone.0256060)

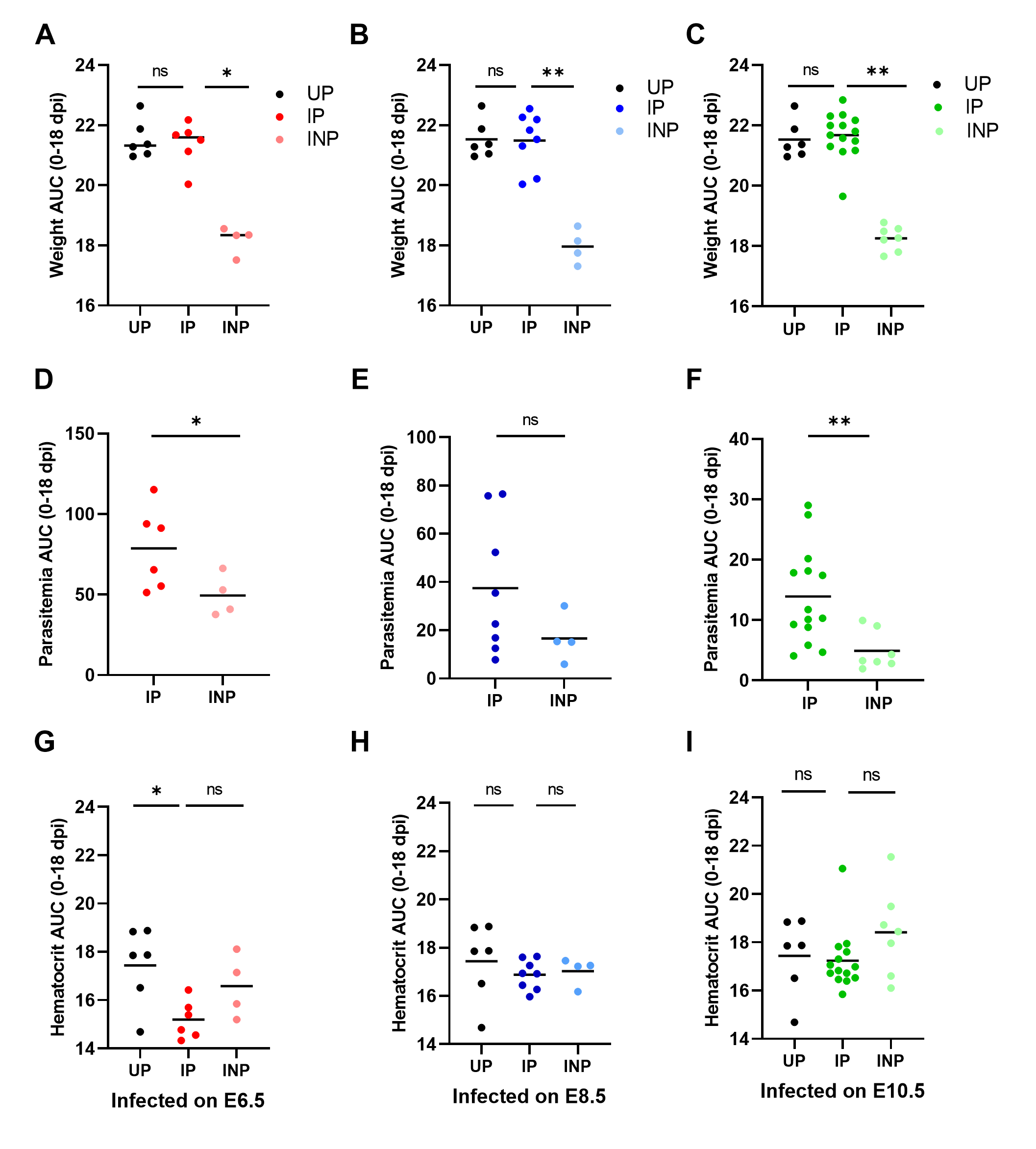

Supplement: S1 Fig — (A-C) Area under the curve (AUC) was calculated for uninfected pregnant (UP), infected pregnant (IP) and infected non-pregnant (INP) mice belonging to the E6.5 infection group (A, D, G; red), E8.5 infection group (B, E, H; blue), or E10.5 infection group (C, F, I; green) in an observational study. No statistical differences are observed in weight change over time between UP and IP animals. (D-F) Parasitemia AUC is higher in some IP animals compared to INP counterparts. (G-I) Hematocrit AUC was statistically different in the E6.5 infection group only. Groups were compared either by using a Kruskal-Wallis test or unpaired t-test with Welch’s correction (for parasitemia AUC). **P ≤ 0.005, *P < 0.05; ns = not significant, P > 0.05. (TIF) [file pone.0256060.s001.tif]

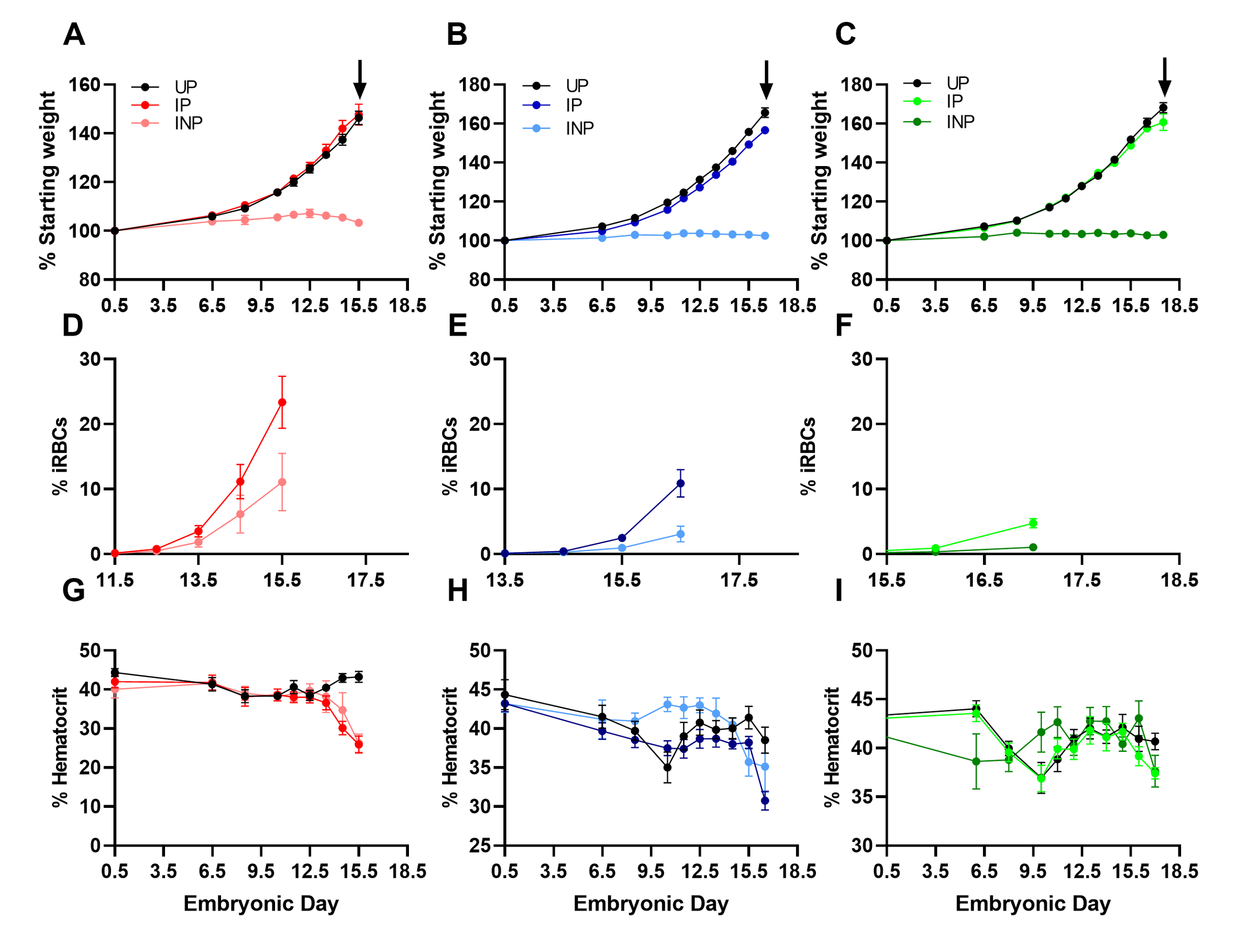

Supplement: S2 Fig — (A-C) Percent starting weight and (D-F) parasitemia (% IRBCs) are presented for infected pregnant (IP), uninfected pregnant (UP), and infected non-pregnant (INP) groups. All mice were sacrificed one day prior to expected preterm delivery (arrows indicate time of sacrifice) and tissues were collected for further studies. (A, D, G) E6.5 infection group, euthanized on E15.5 (red): IP, n = 9; UP, n = 9; and INP, n = 4. (B, E, H) E8.5 infection group, euthanized on E16.5 (blue): IP, n = 15; UP, n = 12; INP, n = 7. (C, F, I) E10.5 infection group, euthanized on E17.5 (green): IP, n = 12; UP, n = 10; INP, n = 6. (TIF) [file pone.0256060.s002.tif]

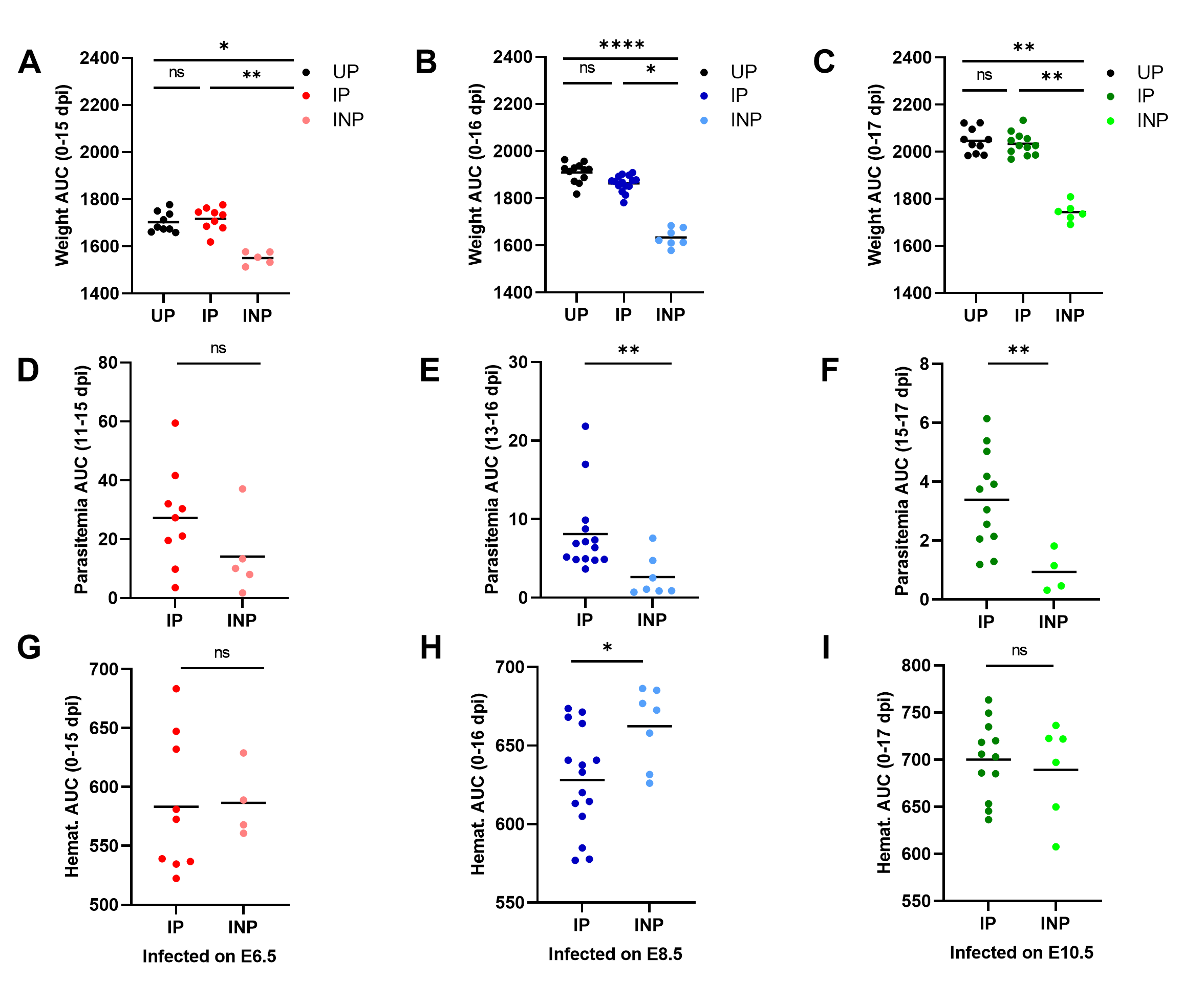

Supplement: S3 Fig — Area under the curve (AUC) was calculated for uninfected pregnant (UP), infected pregnant (IP) and infected non-pregnant (INP) mice belonging to the E6.5 infection group (A, D, G; red), E8.5 infection group (B, E, H; blue), or E10.5 infection group (C, F, I; green) for serial sacrifice experiments. (A-C) No statistical differences are observed in weight change over time between UP and IP animals. (D-F) Parasitemia AUC is higher in some IP animals compared to INP counterparts. (G-I) Hematocrit AUC achieved statistical significance between IP and INP mice in the E8.5 infection group only. Groups were compared either by using a Kruskal-Wallis test or unpaired t-test with Welch’s correction (for parasitemia AUC). ****P < 0.0001, **P < 0.005, *P < 0.05; ns = not significant, P > 0.05. (TIF) [file pone.0256060.s003.tif]

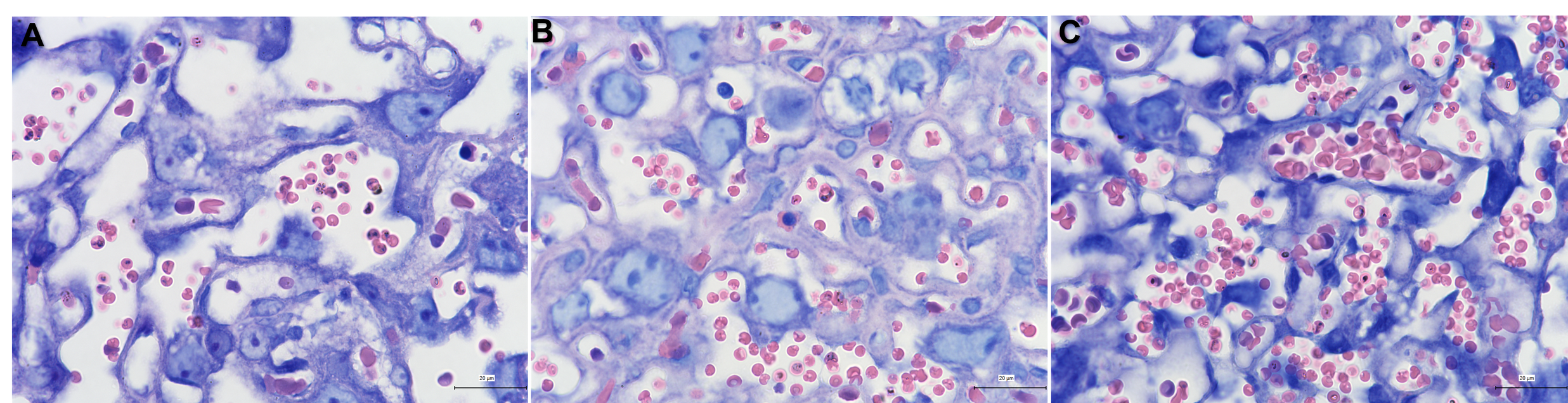

Supplement: S4 Fig — (A-C) micrographs taken with 100x objective lens of Giemsa-stained placentae from infected pregnant (IP) dams sacrificed one day prior to preterm delivery on E15.5, E16.5, and E17.5, respectively. (TIF) [file pone.0256060.s004.tif]

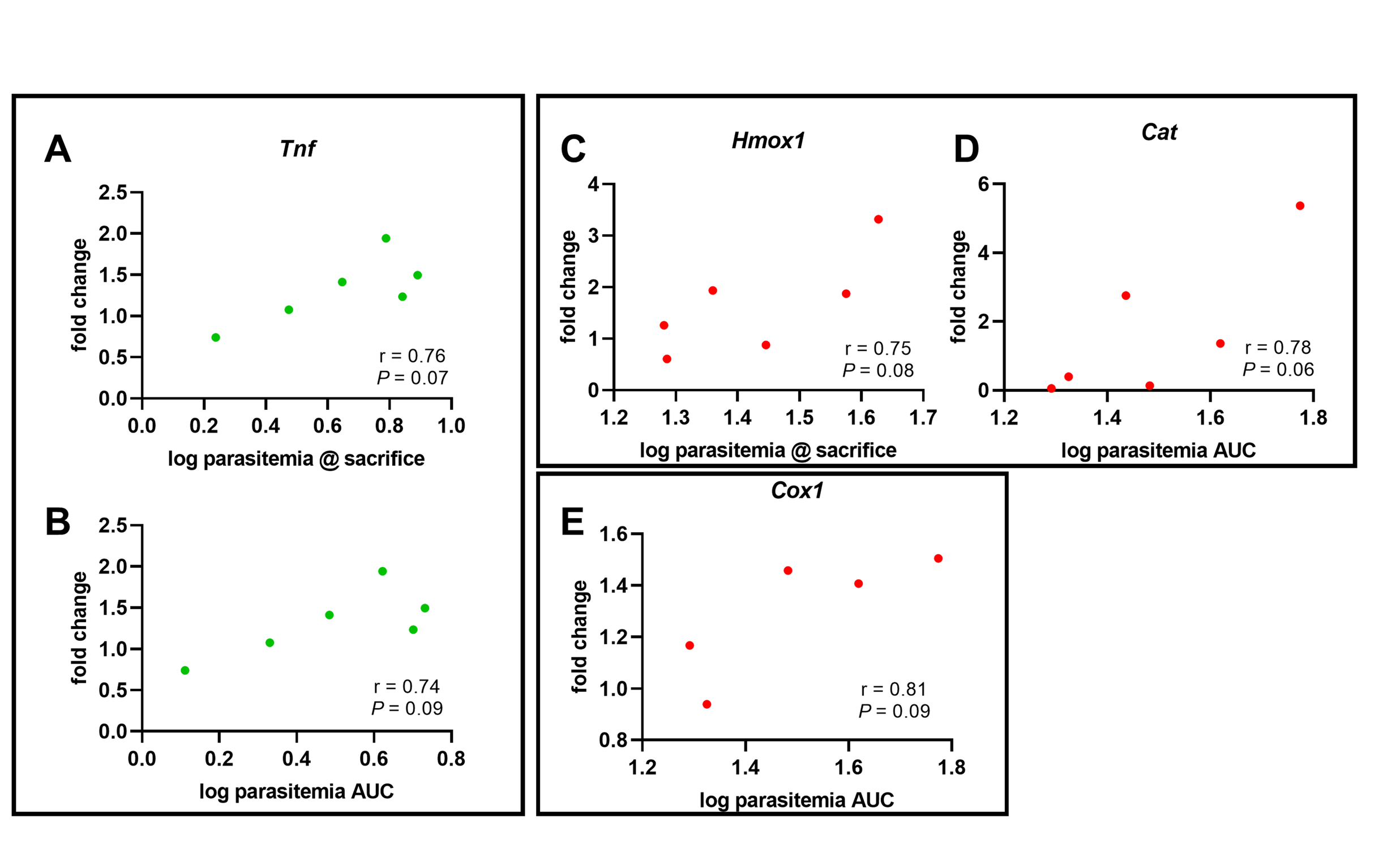

Supplement: S5 Fig — Mouse mRNA transcript abundance relative to peripheral parasitemia at the time of sacrifice or parasitemia AUC in placenta collected one day before expected preterm birth. (A-B) Tnf transcripts tended to be correlated with parasitemia in the E10.5 infection group. (C-D) Cat and Hmox1 transcripts tended to be positively correlated with parasitemia in the E6.5 infection group and (E) Cox1 transcripts tended to have a positive correlation with parasitemia AUC. (TIF) [file pone.0256060.s005.tif]

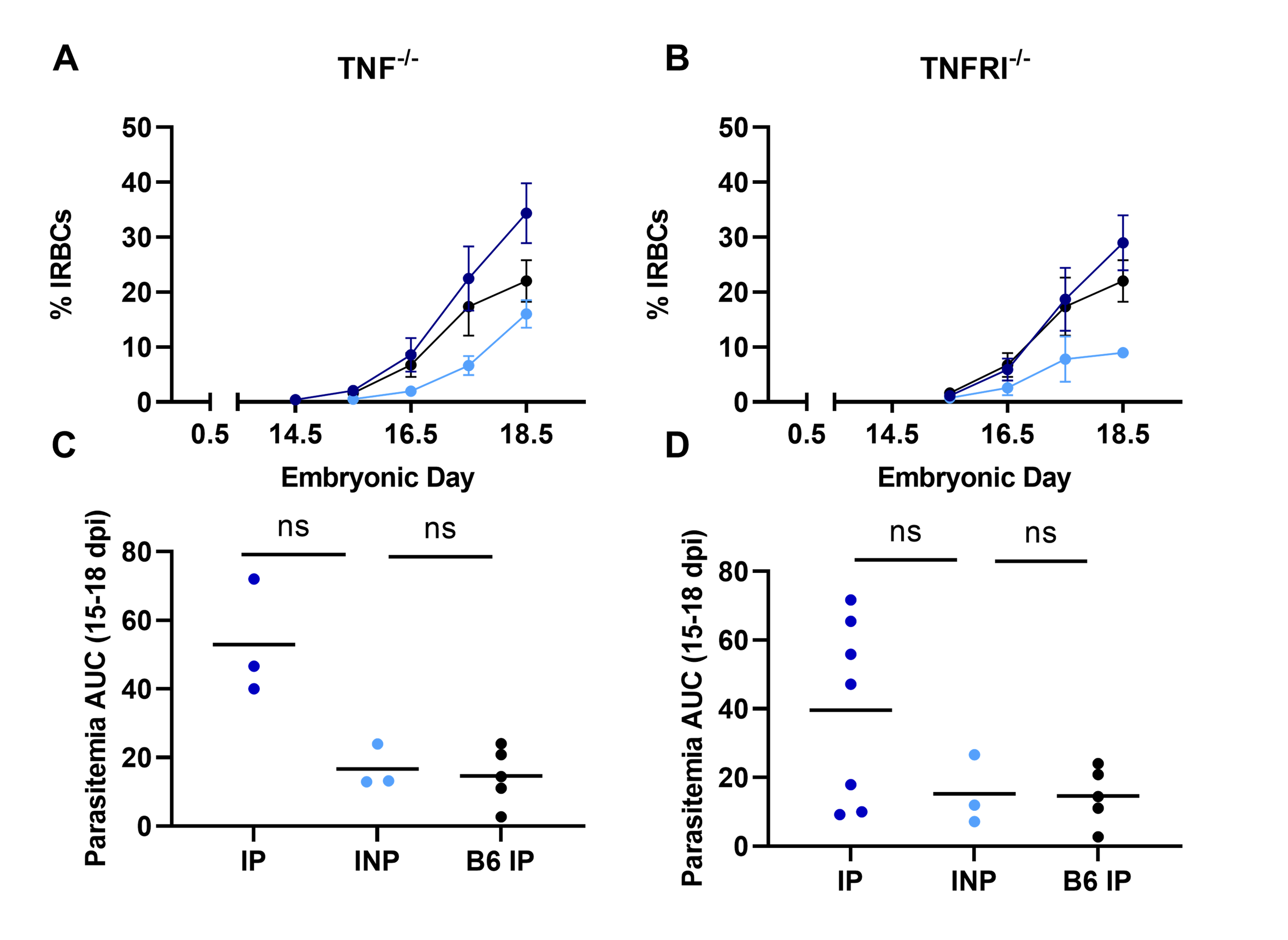

Supplement: S6 Fig — (A) Parasitemia (% IRBCs) in TNF-/- and (B) TNFRI-/- mice. (C-D) Area under the curve (AUC) analysis do not show a statistically significant increase in parasitemia between IP versus INP groups for both strains. TNF-/- IP: n = 3, INP n = 3; TNFRI-/- IP: n = 7, INP n = 3; ns = not significant, P > 0.05. (TIF) [file pone.0256060.s006.tif]
