# Supplementary material for: A novel murine model of post-implantation malaria-induced preterm birth
Source: PLoS One. 2022 Mar 21;17(3):e0256060. doi: 10.1371/journal.pone.0256060 (PMC8936457; doi:10.1371/journal.pone.0256060)
Supplement: S1 Table — No differences were observed in pup viability between infected pregnant (IP) and uninfected pregnant (UP) dams across all infection groups. Statistical significance determined via proportional analysis tested by chi-square. (DOCX) [file pone.0256060.s007.docx]

**S1 Table. Pup viability between infected and uninfected dams sacrificed one day prior to expected preterm delivery.**

| **Infection group** | **Total # pups** | **# non-viable pups** | **% viable** | ***P* value** |
| --- | --- | --- | --- | --- |
| **E6.5 IP** | 63 | 1 | 98.4% | *NS* |
| **E6.5 UP** | 70 | 4 | 94.2% |  |
| **E8.5 IP** | 125 | 5 | 96.0% |  |
| **E8.5 UP** | 100 | 1 | 99.0% |  |
| **E10.5 IP** | 90 | 3 | 96.6% |  |
| **E10.5 UP** | 82 | 2 | 97.5% |  |

No differences were observed in pup viability between infected pregnant (IP) and uninfected pregnant (UP) dams across all infection groups. Statistical significance determined via proportional analysis tested by chi-square.
