# Supplementary material for: A novel murine model of post-implantation malaria-induced preterm birth
Source: PLoS One. 2022 Mar 21;17(3):e0256060. doi: 10.1371/journal.pone.0256060 (PMC8936457; doi:10.1371/journal.pone.0256060)
Supplement: S2 Table — Mouse-specific forward (FP) and reverse (RP) primers used in quantitative real-time PCR for the amplification of mRNA transcripts associated with inflammation, parturition, antioxidant activity, and reference (Ubc) genes. (DOCX) [file pone.0256060.s008.docx]

**S2 Table. Primer sequences for qPCR targets.**

| Target | Forward and Reverse Primers (5’ to 3’) |
| --- | --- |
| *Ubc* | FP: CAGTGTTACCACCAAGAAGGT |
|  | RP: GAAAACTAAGACACCTCCCCCA |
| *Ifng* | FP: AGCAAGGCGAAAAAGGATGC |
|  | RP: CTTCCTGAGGCTGGATTCCG |
| *Tnf* | FP: ATCGGTCCCCAAAGGGATGA |
|  | RP: GTCTTTGAGATCCATGCCGT |
| *Il10* | FP: CTGAAGACCCTCAGGATGCG |
|  | RP: TGGCCTTGTAGACACCTTGG |
| *Il1b* | FP: TGAAGTTGACGGACCCCAAA |
|  | RP: TGATACTGCCTGCCTGAAGC |
| *Cox1* | FP: GATTGTACTCGCACGGGCTAC |
|  | RP: GGATAAGGTTGGACCGCACT |
| *Cox2* | FP: TGTGACTGTACCCGGACTGG |
|  | RP: TGCACATTGTAAGTAGGTGGAC |
| *Sod1* | FP: GGAACCATCCACTTCGAGCA |
|  | RP: CTGCACTGGTACAGCCTTGT |
| *Sod2* | FP: GGACCCATTGCAAGGAACAAC |
|  | RP: TGAGTGAGGTTTCACTTCTTGC |
| *Sod3* | FP: GCAACTCAGAGGCTCTTCCTC |
|  | RP: CCCCTGGATTTGACATGGTGA |
| *Nrf2* | FP: AGCCAGCTGACCTCCTTAGA |
|  | RP: AGTGACTGACTGATGGCAGC |
| *Cat* | FP: CACTGACGAGATGGCACACT |
|  | RP: TGTGGAGAATCGAACGGCAA |
| *Hmox1* | FP: CATAGCCCGGAGCCTGAATC |
|  | RP: AGCATTCTCGGCTTGGATGT |

Mouse-specific forward (FP) and reverse (RP) primers used in quantitative real-time PCR for the amplification of mRNA transcripts associated with inflammation, parturition, antioxidant activity, and reference (*Ubc*) genes.
