# Supplementary material for: A novel murine model of post-implantation malaria-induced preterm birth
Source: PLoS One. 2022 Mar 21;17(3):e0256060. doi: 10.1371/journal.pone.0256060 (PMC8936457; doi:10.1371/journal.pone.0256060)
Supplement: S3 Table — Analysis performed with proc reg for dichotomous (status) and continuous (parasitemia) and variables and proc glm for categorical variables (sacrifice day). Parasitemia was log10-transformed for the analysis. Dashes indicate that E15.5 is the reference value. Sample sizes for the analysis are as follows: E15.5 IP, n = 4; E15.5 UP, n = 4; E16.5 IP, n = 11; E16.5 UP, n = 4; E17.5 IP, n = 6; E17.5 UP, n = 3. (DOCX) [file pone.0256060.s009.docx]

**S3 Table. Univariate logistic regression analysis of inflammatory and parturition-associated transcript expression and day of sacrifice**

|  | *Ifng* | | *Tnf* | | *Il1b* | | *Il10* | | *Cox1* | | *Cox2* | |
| --- | --- | --- | --- | --- | --- | --- | --- | --- | --- | --- | --- | --- |
|  | Co-effi  cient; SEM | P | Co-effi  cient; SEM | P | Co-effi  cient; SEM | P | Co-effi  cient; SEM | P | Co-effi  cient; SEM | P | Co-effi  cient; SEM | P |
| **Categorical variables** | | | | | | | | | | | | |
| Intercept | 1.09; 0.30 | 0.001 | 1.12; 0.26 | 0.0001 | 1.15; 0.21 | ˂.0001 | 1.38; 0.39 | 0.001 | 1.21; 0.21 | ˂.0001 | 1.24; 0.77 | 0.12 |
| E15.5 sacrifice | - | - | - | - | - | - | - | - | - | - | - | - |
| E16.5 sacrifice | 2.29; 0.40 | 0.004 | 2.14; 0.33 | 0.004 | 1.92; 0.26 | 0.005 | 2.68; 0.51 | 0.01 | 1.29; 0.08 | 0.74 | 5.50; 0.97 | ˂.0001 |
| E17.5 sacrifice | 1.69; 0.43 | 0.17 | 1.25; 0.37 | 0.71 | 1.20; 0.29 | 0.84 | 1.09; 0.57 | 0.61 | 0.997; 0.29 | 0.47 | 1.15; 1.06 | 0.93 |
|  |  |  |  |  |  |  |  |  |  |  |  |  |
| Intercept | 1.07; 0.26 | 0.0002 | 1.13; 0.25 | ˂.0001 | 1.08; 0.19 | ˂.0001 | 1.07; 0.38 | 0.007 | 0.925; 0.17 | ˂.0001 | 1.38; 0.76 | 0.07 |
| Status (IP) | 2.17; 0.33 | 0.002 | 1.84; 0.31 | 0.02 | 1.73; 0.23 | 0.009 | 2.27; 0.47 | 0.01 | 1.34; 0.21 | 0.05 | 4.25; 0.97 | 0.005 |
| **Continuous variables** | | | | | | | | | | | | |
| Intercept | 1.16; 0.27 | 0.0002 | 1.17; 0.20 | ˂.0001 | 1.10; 0.16 | ˂.0001 | 1.07; 0.25 | 0.0002 | 0.897; 0.16 | ˂.0001 | 1.49; 0.65 | 0.03 |
| Placental parasitemia | 1.94; 0.28 | 0.009 | 1.74; 0.20 | 0.01 | 1.68; 0.16 | 0.001 | 1.88; 0.25 | 0.003 | 1.22; 0.16 | 0.05 | 3.69; 0.70 | 0.004 |
|  |  |  |  |  |  |  |  |  |  |  |  |  |
| Intercept | 1.38; 0.26 | ˂.0001 | 1.30; 0.24 | ˂.0001 | 1.20; 0.18 | ˂.0001 | 1.32; 0.36 | 0.0008 | 0.940; 0.16 | ˂.0001 | 1.84; 0.72 | 0.02 |
| Peripheral parasitemia | 1.98; 0.31 | 0.05 | 1.74; 0.28 | 0.12 | 1.65; 0.21 | 0.03 | 2.13; 0.41 | 0.06 | 1.34; 0.18 | 0.03 | 4.00; 0.89 | 0.02 |
|  |  |  |  |  |  |  |  |  |  |  |  |  |
| Intercept | 1.89; 0.17 | ˂.0001 | 1.67; 0.16 | ˂.0001 | 1.58; 0.12 | ˂.0001 | 1.98; 0.24 | ˂.0001 | 1.25; 0.10 | ˂.0001 | 3.58; 0.51 | ˂.0001 |
| Peripheral parasitemia AUC | 2.00; 0.037 | 0.007 | 1.74; 0.034 | 0.04 | 1.64; 0.026 | 0.01 | 2.11; 0.051 | 0.02 | 1.30; 0.023 | 0.04 | 3.88; 0.10 | 0.008 |
