# Supplementary material for: A novel murine model of post-implantation malaria-induced preterm birth
Source: PLoS One. 2022 Mar 21;17(3):e0256060. doi: 10.1371/journal.pone.0256060 (PMC8936457; doi:10.1371/journal.pone.0256060)
Supplement: S4 Table — Analysis performed with proc reg for continuous (parasitemia) and dichotomous (status) variables and proc glm for categorical variables (sacrifice day). *Parasitemias were log10-transformed for the analysis. Dashes indicate that E15.5 is the reference value. Sample sizes for the analysis are as follows: E15.5 IP, n = 4; E15.5 UP, n = 4; E16.5 IP, n = 11; E16.5 UP, n = 4; E17.5 IP, n = 6; E17.5 UP, n = 3. (DOCX) [file pone.0256060.s010.docx]

**S4 Table. Univariate logistic regression analysis of antioxidant transcript expression and day of sacrifice**

|  | *Nrf2* | | *Sod1* | | *Sod2* | | *Sod3* | | *Cat* | | *Hmox1* | |
| --- | --- | --- | --- | --- | --- | --- | --- | --- | --- | --- | --- | --- |
|  | Co-effi  cient; SEM | P | Co-effi  cient; SEM | P | Co-effi  cient; SEM | P | Co-effi  cient; SEM | P | Co-effi  cient; SEM | P | Co-effi  cient; SEM | P |
| **Categorical variables** | | | | | | | | | | | | |
| Intercept | 1.20; 0.46 | 0.01 | 1.23; 0.60 | 0.04 | 1.14; 0.29 | 0.004 | 0.94; 0.12 | ˂.0001 | 1.36; 0.65 | 0.04 | 1.32; 0.17 | ˂.0001 |
| E15.5 sacrifice | - | - | - | - | - | - | - | - | - | - | - | - |
| E16.5 sacrifice | 3.73; 0.60 | 0.0002 | 3.99; 0.77 | 0.001 | 2.10; 0.37 | 0.01 | 0.825; 0.15 | 0.44 | 4.28; 0.84 | 0.0013 | 1.44; 0.22 | 0.59 |
| E17.5 sacrifice | 1.32; 0.66 | 0.86 | 1.54; 0.84 | 0.71 | 0.915; 0.41 | 0.59 | 0.89; 0.17 | 0.75 | 1.29; 0.92 | 0.94 | 1.12; 0.25 | 0.44 |
|  |  |  |  |  |  |  |  |  |  |  |  |  |
| Intercept | 1.33; 0.47 | 0.007 | 1.29; 0.56 | 0.02 | 1.05; 0.27 | 0.0004 | 0.940; 0.10 | ˂.0001 | 1.25; 0.61 | 0.05 | 1.07; 0.14 | ˂.0001 |
| Status (IP) | 2.96; 0.59 | 0.009 | 3.29; 0.70 | 0.007 | 1.77; 0.72 | 0.04 | 0.84; 0.13 | 0.47 | 3.46; 0.78 | 0.007 | 1.48; 0.41 | 0.03 |
| **Continuous variables** | | | | | | | | | | | | |
| Intercept | 1.26; 0.40 | 0.002 | 1.33; 0.39 | 0.002 | 1.05; 0.23 | 0.0001 | 0.927; 0.088 | ˂.0001 | 1.24; 0.49 | 0.01 | 1.02; 0.14 | ˂.0001 |
| Placental parasitemia | 2.65; 0.42 | 0.005 | 2.55; 0.40 | 0.005 | 1.59; 0.24 | 0.03 | 0.756; 0.09 | 0.07 | 2.88; 0.51 | 0.003 | 1.48; 0.15 | 0.004 |
|  |  |  |  |  |  |  |  |  |  |  |  |  |
| Intercept | 1.56; 0.44 | 0.001 | 1.63; 0.53 | 0.004 | 1.13; 0.25 | ˂.0001 | 0.956; 0.095 | ˂.0001 | 1.53; 0.58 | 0.01 | 1.02; 0.13 | ˂.0001 |
| Peripheral parasitemia | 2.81; 0.53 | 0.02 | 3.08; 0.64 | 0.02 | 1.72; 0.30 | 0.05 | 0.834; 0.11 | 0.29 | 3.29; 0.69 | 0.01 | 1.50; 0.15 | 0.002 |
|  |  |  |  |  |  |  |  |  |  |  |  |  |
| Intercept | 2.57; 0.30 | ˂.0001 | 2.81; 0.36 | ˂.0001 | 1.61; 0.18 | ˂.0001 | 0.865; 0.067 | ˂.0001 | 2.95; 0.40 | ˂.0001 | 1.40; 0.052 | ˂.0001 |
| Peripheral parasitemia AUC | 2.74; 0.066 | 0.01 | 3.02; 0.078 | 0.013 | 1.68; 0.038 | 0.047 | 0.854; 0.015 | 0.42 | 3.19; 0.086 | 0.008 | 1.45; 0.019 | 0.012 |
