# Supplementary material for: A novel murine model of post-implantation malaria-induced preterm birth
Source: PLoS One. 2022 Mar 21;17(3):e0256060. doi: 10.1371/journal.pone.0256060 (PMC8936457; doi:10.1371/journal.pone.0256060)
Supplement: S5 Table — Analysis performed with proc glm. Dashes indicate that E15.5 is the reference value; dashes and NA indicate that these parameters were not considered in the analysis. Sample sizes for the analysis are as follows: E15.5 IP, n = 4; E16.5 IP, n = 11; E17.5 IP, n = 6. (DOCX) [file pone.0256060.s011.docx]

**S5 Table. Multivariate logistic regression analysis of inflammatory and parturition-associated transcript expression and day of sacrifice**

|  | *Ifng* | | *Tnf* | | *Il1b* | | *Il10* | | *Cox1* | | *Cox2* | |
| --- | --- | --- | --- | --- | --- | --- | --- | --- | --- | --- | --- | --- |
|  | Co-effi  cient; SEM | P | Co-effi  cient; SEM | P | Co-effi  cient; SEM | P | Co-effi  cient; SEM | P | Co-effi  cient; SEM | P | Co-effi  cient; SEM | P |
| **Categorical variables** | | | | | | | | | | | | |
| Intercept | 0.562; 0.32 | 0.09 | 0.809; 0.30 | 0.009 | 0.867; 0.23 | 0.0006 | 0.834; 0.44 | 0.07 | 0.982; 0.24 | 0.0002 | 0.165; 0.80 | 0.84 |
| Status (IP) | 1.53; 0.31 | 0.004 | 1.38; 0.30 | 0.06 | 1.89; 0.22 | 0.027 | 1.83; 0.43 | 0.03 | 1.40; 0.22 | 0.06 | 2.31; 0.75 | 0.007 |
| E15.5 sacrifice | - | - | - | - | - | - | - | - | - | - | - | - |
| E16.5 sacrifice | 1.59; 0.32 | 0.008 | 1.73; 0.33 | 0.009 | 1.37; 0.26 | 0.01 | 1.95; 0.57 | 0.03 | 1.00; 0.25 | 0.93 | 4.00; 0.90 | 0.0002 |
| E17.5 sacrifice | 1.16; 0.40 | 0.13 | 0.915; 0.36 | 0.77 | 0.924; 0.27 | 0.83 | 0.492; 0.54 | 0.53 | 0.717; 0.29 | 0.37 | -0.0138; 0.972 | 0.85 |
| **Continuous variables** | | | | | | | | | | | | |
| Placental parasitemia | NA | - | NA | - | NA | - | NA | - | NA | - | NA | - |
| Peripheral parasitemia | NA | - | NA | - | NA | - | NA | - | NA | - | NA | - |
| Peripheral parasitemia AUC | NA | - | NA | - | NA | - | NA | - | NA | - | NA | - |
