# Supplementary material for: A novel murine model of post-implantation malaria-induced preterm birth
Source: PLoS One. 2022 Mar 21;17(3):e0256060. doi: 10.1371/journal.pone.0256060 (PMC8936457; doi:10.1371/journal.pone.0256060)
Supplement: S6 Table — Analysis performed with proc glm. Dashes indicate that E15.5 is the reference value; dashes and NA indicate that these parameters were not considered in the analysis. Sample sizes for the analysis are as follows: E15.5 IP, n = 4; E16.5 IP, n = 11; E17.5 IP, n = 6. (DOCX) [file pone.0256060.s012.docx]

**S6 Table. Multivariate logistic regression analysis of antioxidant transcript expression and day of sacrifice**

|  | *Nrf2* | | *Sod1* | | *Sod2* | | *Sod3* | | *Cat* | | *Hmox1* | |
| --- | --- | --- | --- | --- | --- | --- | --- | --- | --- | --- | --- | --- |
|  | Co-effi  cient; SEM | P | Co-effi  cient; SEM | P | Co-effi  cient; SEM | P | Co-effi  cient; SEM | P | Co-effi  cient; SEM | P | Co-effi  cient; SEM | P |
| **Categorical variables** | | | | | | | | | | | | |
| Intercept | 0.517; 0.50 | 0.31 | 0.347; 0.65 | 0.60 | 0.837; 0.34 | 0.01 | 0.992; 014 | ˂.0001 | 0.392; 0.71 | 0.58 | 1.16; 0.20 | ˂.0001 |
| Status (IP) | 1.77; 0.48 | 0.01 | 1.97; 0.61 | 0.01 | 1.40; 0.34 | 0.08 | 0.911; 0.14 | 0.56 | 2.17; 0.67 | 0.01 | 1.50; 0.19 | 0.05 |
| E15.5 sacrifice | - | - | - | - | - | - | - | - | - | - | - | - |
| E16.5 sacrifice | 2.84; 0.56 | 0.0002 | 2.86; 0.72 | 0.001 | 1.71; 0.27 | 0.02 | 0.883; 0.16 | 0.50 | 3.03; 0.79 | 0.002 | 1.18; 0.20 | 0.80 |
| E17.5 sacrifice | 0.635; 0.61 | 0.85 | 0.656; 0.78 | 0.70 | 0.612; 0.42 | 0.58 | 0.944; 0.18 | 0.79 | 0.328; 0.86 | 0.94 | 0.924; 0.24 | 0.43 |
| **Continuous variables** | | | | | | | | | | | | |
| Placental parasitemia | NA | - | NA | - | NA | - | NA | - | NA | - | NA | - |
| Peripheral parasitemia | NA | - | NA | - | NA | - | NA | - | NA | - | NA | - |
| Peripheral parasitemia AUC | NA | - | NA | - | NA | - | NA | - | NA | - | NA | - |
