# Supplementary material for: A novel murine model of post-implantation malaria-induced preterm birth
Source: PLoS One. 2022 Mar 21;17(3):e0256060. doi: 10.1371/journal.pone.0256060 (PMC8936457; doi:10.1371/journal.pone.0256060)
Supplement: S7 Table — Analysis performed with proc glm. Dashes indicate that E15.5 is the reference value; dashes and NA indicate that these parameters were not considered in the analysis. Sample sizes for the analysis are as follows: E15.5 IP, n = 4; E16.5 IP, n = 11; E17.5 IP, n = 6. (DOCX) [file pone.0256060.s013.docx]

**S7 Table.** **Multivariate logistic regression analysis of inflammatory and parturition-associated transcript expression and day of sacrifice**

|  | *Ifng* | | *Tnf* | | *Il1b* | | *Il10* | | *Cox1* | | *Cox2* | |
| --- | --- | --- | --- | --- | --- | --- | --- | --- | --- | --- | --- | --- |
|  | Co-effi  cient; SEM | P | Co-effi  cient; SEM | P | Co-effi  cient; SEM | P | Co-effi  cient; SEM | P | Co-effi  cient; SEM | P | Co-effi  cient; SEM | P |
| **Categorical variables** | | | | | | | | | | | | |
| Intercept | 1.22; 0.28 | 0.0001 | 1.19; 0.25 | ˂.0001 | 1.22; 0.20 | ˂.0001 | 1.51; 0.38 | 0.0003 | 1.27; 0.20 | ˂.0001 | 1.64; 0.72 | ˂.0001 |
| Status (IP) | NA | - | NA | - | NA | - | NA | - | NA | - | NA | - |
| E15.5 sacrifice | - | - | - | - | - | - | - | - | - | - | - | - |
| E16.5 sacrifice | 2.34; 0.37 | 0.004 | 2.17; 0.33 | 0.005 | 0.730; 0.25 | 0.007 | 3.94; 0.50 | 0.02 | 1.33; 0.25 | 0.80 | 5.66; 0.90 | ˂.0001 |
| E17.5 sacrifice | 1.94; 0.40 | 0.07 | 1.38; 0.36 | 0.61 | 1.34; 0.28 | 0.65 | 1.32; 0.54 | 0.71 | 1.07; 0.29 | 0.50 | 1.73; 0.98 | 0.92 |
| **Continuous variables** | | | | | | | | | | | | |
| Placental parasitemia | NA | - | NA | - | NA | - | NA | - | NA | - | NA | - |
| Peripheral parasitemia | NA | - | NA | - | NA | - | NA | - | NA | - | NA | - |
| Peripheral parasitemia AUC | 1.31; 0.035 | 0.007 | 1.25; 0.031 | 0.07 | 1.27; 0.024 | 0.03 | 1.61; 0.047 | 0.04 | 1.31; 0.024 | 0.06 | 1.58; 0.083 | 0.009 |
