# Supplementary material for: A novel murine model of post-implantation malaria-induced preterm birth
Source: PLoS One. 2022 Mar 21;17(3):e0256060. doi: 10.1371/journal.pone.0256060 (PMC8936457; doi:10.1371/journal.pone.0256060)
Supplement: S8 Table — Analysis performed with proc glm. Dashes indicate that E15.5 is the reference value; dashes and NA indicate that these parameters were not considered in the analysis. Sample sizes for the analysis are as follows: E15.5 IP, n = 4; E15.5 UP, n = 4; E16.5 IP, n = 11; E16.5 UP, n = 4; E17.5 IP, n = 6; E17.5 UP, n = 3. (DOCX) [file pone.0256060.s014.docx]

**S8 Table. Multivariate logistic regression analysis of antioxidant transcript expression and day of sacrifice**

|  | *Nrf2* | | *Sod1* | | *Sod2* | | *Sod3* | | *Cat* | | *Hmox1* | |
| --- | --- | --- | --- | --- | --- | --- | --- | --- | --- | --- | --- | --- |
|  | Co-effi  cient; SEM | P | Co-effi  cient; SEM | P | Co-effi  cient; SEM | P | Co-effi  cient; SEM | P | Co-effi  cient; SEM | P | Co-effi  cient; SEM | P |
| **Categorical variables** | | | | | | | | | | | | |
| Intercept | 1.38; 0.43 | 0.003 | 1.46; 0.56 | 0.01 | 1.22; 0.29 | 0.02 | 0.934; 0.13 | ˂.0001 | 1.62; 0.61 | 0.01 | 1.39; 0.17 | ˂.0001 |
| Status (IP) | NA | - | NA | - | NA | - | NA | - | NA | - | NA | - |
| E15.5 sacrifice | - | - | - | - | - | - | - | - | - | - | - | - |
| E16.5 sacrifice | 3.82; 0.57 | 0.0001 | 4.12; 0.72 | 0.0007 | 2.15; 0.37 | 0.01 | 0.819; 0.16 | 0.47 | 4.42; 0.78 | 0.0009 | 1.48; 0.22 | 0.67 |
| E17.5 sacrifice | 1.67; 0.62 | 0.64 | 1.99; 0.79 | 0.50 | 1.07; 0.41 | 0.72 | 0.873; 0.18 | 0.73 | 1.80; 0.86 | 0.83 | 1.26; 0.24 | 0.58 |
| **Continuous variables** | | | | | | | | | | | | |
| Placental parasitemia | NA | - | NA | - | NA | - | NA | - | NA | - | NA | - |
| Peripheral parasitemia | NA | - | NA | - | NA | - | NA | - | NA | - | NA | - |
| Peripheral parasitemia AUC | 1.52; 0.53 | 0.01 | 1.63; 0.068 | 0.01 | 1.28; 0.035 | 0.08 | 0.922; 0.015 | 0.47 | 1.81; 0.073 | 0.001 | 1.44; 0.020 | 0.02 |
