# Supplementary material for: A novel murine model of post-implantation malaria-induced preterm birth
Source: PLoS One. 2022 Mar 21;17(3):e0256060. doi: 10.1371/journal.pone.0256060 (PMC8936457; doi:10.1371/journal.pone.0256060)
Supplement: S9 Table — Analysis performed with proc glm. Dashes indicate that E17.5 is the reference value; dashes and NA indicate that these parameters were not considered in the analysis. Sample sizes for the analysis are as follows: E15.5 IP, n = 2; E16.5 IP, n = 7; E17.5 IP, n = 3. (DOCX) [file pone.0256060.s015.docx]

**S9 Table. Multivariate logistic regression analysis of inflammatory and parturition-associated transcript expression and day of sacrifice**

|  | *Ifng* | | *Tnf* | | *Il1b* | | *Il10* | | *Cox1* | | *Cox2* | |
| --- | --- | --- | --- | --- | --- | --- | --- | --- | --- | --- | --- | --- |
|  | Co-effi  cient; SEM | P | Co-effi  cient; SEM | P | Co-effi  cient; SEM | P | Co-effi  cient; SEM | P | Co-effi  cient; SEM | P | Co-effi  cient; SEM | P |
| **Categorical variables** | | | | | | | | | | | | |
| Intercept | 1.30; 0.36 | 0.002 | 1.06; 0.29 | 0.001 | 1.12; 0.22 | ˂.0001 | 0.828; 0.36 | 0.03 | 0.811; 0.25 | 0.004 | 0.471; 0.80 | 0.57 |
| Status (IP) | NA | - | NA | - | NA | - | NA | - | NA | - | NA | - |
| E15.5 sacrifice | 0.620; 0.46 | 0.17 | 0.906; 0.37 | 0.68 | 0.885; 0.29 | 0.42 | 0.998; 0.46 | 0.71 | 1.04; 0.32 | 0.48 | 0.433; 1.08 | 0.97 |
| E16.5 sacrifice | 1.51; 0.46 | 0.65 | 1.53; 0.352 | 0.20 | 1.28; 0.28 | 0.57 | 1.40; 0.45 | 0.22 | 0.866; 0.28 | 0.85 | 3.39; 0.10 | 0.007 |
| E17.5 sacrifice | - | - | - | - | - | - | - | - | - | - | - | - |
| **Continuous variables** | | | | | | | | | | | | |
| Placental parasitemia | 2.06; 0.28 | 0.01 | 1.58; 0.21 | 0.02 | 1.69; 0.17 | 0.003 | 1.60; 0.26 | 0.007 | 1.14; 0.17 | 0.07 | 2.24; 0.62 | 0.008 |
| Peripheral parasitemia | NA | - | NA | - | NA | - | NA | - | NA | - | NA | - |
| Peripheral parasitemia AUC | NA | - | NA | - | NA | - | NA | - | NA | - | NA | - |
