# Supplementary material for: A novel murine model of post-implantation malaria-induced preterm birth
Source: PLoS One. 2022 Mar 21;17(3):e0256060. doi: 10.1371/journal.pone.0256060 (PMC8936457; doi:10.1371/journal.pone.0256060)
Supplement: S10 Table — Analysis performed with proc glm. Dashes indicate that E17.5 is the reference value; dashes and NA indicate that these parameters were not considered in the analysis. Sample sizes for the analysis are as follows: E15.5 IP, n = 2; E16.5 IP, n = 7; E17.5 IP, n = 3. (DOCX) [file pone.0256060.s016.docx]

**S10 Table. Multivariate logistic regression analysis of antioxidant transcript expression and day of sacrifice**

|  | *Nrf2* | | *Sod1* | | *Sod2* | | *Sod3* | | *Cat* | | *Hmox1* | |
| --- | --- | --- | --- | --- | --- | --- | --- | --- | --- | --- | --- | --- |
|  | estimate; SEM | P | estimate; SEM | P | estimate; SEM | P | estimate; SEM | P | estimate; SEM | P | estimate; SEM | P |
| **Categorical variables** | | | | | | | | | | | | |
| Intercept | 0.919; 0.51 | 0.08 | 1.01; 0.51 | 0.05 | 0.782; 0.336 | 0.02 | 0.825; 0.12 | ˂.0001 | 0.771; 0.66 | 0.25 | 0.950; 0.21 | 0.0002 |
| Status (IP) | NA | - | NA | - | NA | - | NA | - | NA | - | NA | - |
| E15.5 sacrifice | 0.640; 0.68 | 0.68 | 0.644; 0.68 | 0.58 | 0.973; 0.44 | 0.67 | 1.05; 0.17 | 0.18 | 0.553; 0.89 | 0.80 | 1.09; 0.28 | 0.61 |
| E16.5 sacrifice | 2.37; 0.64 | 0.03 | 2.19; 0.63 | 0.07 | 1.38; 0.41 | 0.16 | 0.930; 0.16 | 0.51 | 2.24; 0.83 | 0.08 | 1.03; 0.26 | 0.75 |
| E17.5 sacrifice | - | - | - | - | - | - | - | - | - | - | - | - |
| **Continuous variables** | | | | | | | | | | | | |
| Placental parasitemia | 2.05; 0.38 | 0.007 | 2.12; 0.38 | 0.008 | 1.24; 0.25 | 0.07 | 0.634; 0.09 | 0.05 | 2.26; 0.50 | 0.006 | 1.39; 0.16 | 0.009 |
| Peripheral parasitemia | NA | - | NA | - | NA | - | NA | - | NA | - | NA | - |
| Peripheral parasitemia AUC | NA | - | NA | - | NA | - | NA | - | NA | - | NA | - |
